# Dicarbonyl-1κ^2^*C*-μ-chlorido-2:3κ^2^*Cl*:*Cl*-penta­chlorido-2κ^2^*Cl*,3κ^3^*Cl*-[1(η^6^)-toluene]digallium(III)ruthenium(I)(*Ru*—*Ga*)

**DOI:** 10.1107/S2414314624006576

**Published:** 2024-07-09

**Authors:** Danielle Smith, George N. Harakas

**Affiliations:** a PO Box 6949, Radford University, Radford, Virginia 24142, USA; Goethe-Universität Frankfurt, Germany

**Keywords:** crystal structure, ruthenium, gallium, metal–organic

## Abstract

The title compound, [RuGa_2_Cl_6_(C_7_H_8_)(CO)_2_] or [(CO)_2_(GaCl_2_)(η^6^-toluene)Ru]^+^[GaCl_4_]^−^, was isolated from the reaction of Ga_2_Cl_4_ with di­phenyl­silanediol in toluene, followed by the addition of Ru_3_(CO)_12_. The compound contains a ruthenium–gallium metal–metal bond with a length of 2.4575 (2) Å.

## Abstract 2

The title compound is a ruthenium–gallium metal cluster. The ruthenium and gallium have a direct metal–metal bond with a length of 2.4575 (2) Å.

## Graphical abstract

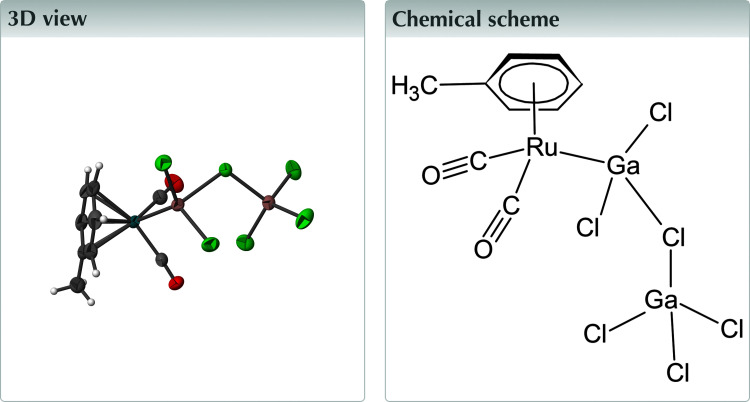

## Structure description

**Figure 1:**
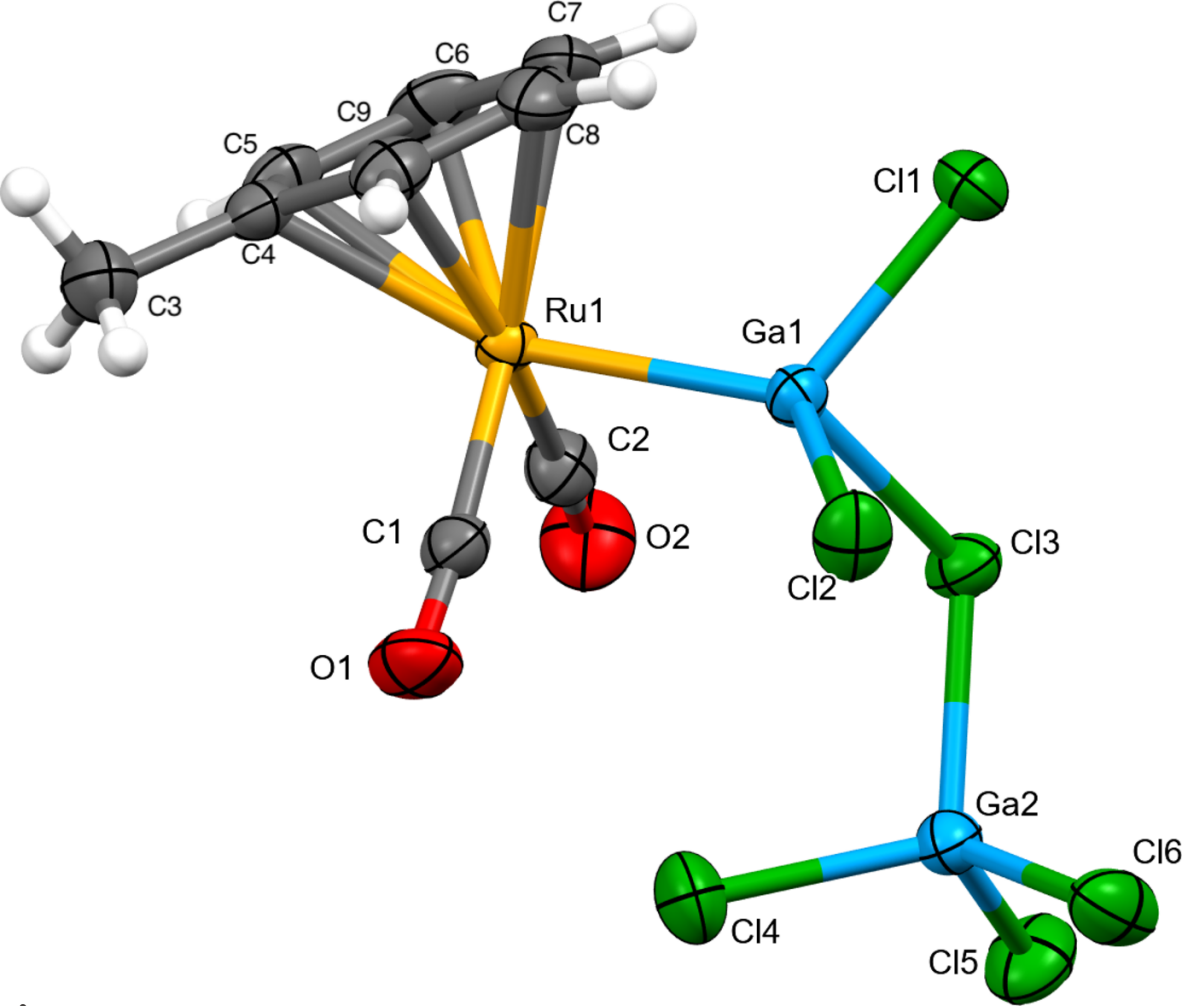
The mol­ecular structure of the title compound. Displacement ellipsoids are drawn at the 50% probability level.

The reaction of Ga_2_Cl_4_ with Ru_3_(CO)_12_ in toluene was demonstrated to produce two ruthenium–gallium metal clusters (Harakas & Whittlesey, 1997 ▸). The title compound (Fig. 1 ▸) was isolated during an attempt to synthesize the ruthenium–gallium di­phenyl­siloxane analogue of the previously reported iron–gallium di­methyl­siloxane cluster (Demmin *et al.*, 2024 ▸).

The title compound can be described as [(CO)_2_(GaCl_2_)(η^6^-toluene) Ru]^+^ [GaCl_4_]^−^. A single positive charge on the ruthenium complex provides a total of 18 electrons for the metal center with [GaCl_4_]^−^ for charge balance. This bonding model is supported by the Ga1—Cl3 bond length of 2.4619 (5) Å, which is significantly longer than the other Ga—Cl bond lengths (Table 1 ▸) observed in the title compound. In contrast, [{CpFe(CO)_2_}Ga(Cl*GaCl_3_)(μ-Cl)]_2_ is described as a Lewis acid–base complex *i.e.* [{CpFe(CO)_2_}GaCl_2_]_2_·2GaCl_3_ (Borovik *et al.*, 1999 ▸). The bond angles for the terminal GaCl_3_ are 112.87, 114.74, and 114.09°, which are all significantly greater than the 109.5° of tetra­hedral geometry. For the title compound, the corresponding angles around Ga2 (Table 1 ▸) are much closer to the ideal tetra­hedral geometry, which is consistent with [GaCl_4_]^−^. In tetra­ethyl ­ammonium tetra­chlorido­gallium (Bolte *et al.*, 2023 ▸), the Cl—Ga—Cl bond angles range from 108.1 to 110.1°. An analogous Lewis acid–base bonding model for the title complex would require a 19 electron ruthenium metal center or that Ga1 carries one formal negative charge, both of which are unlikely.

**Figure 2:**
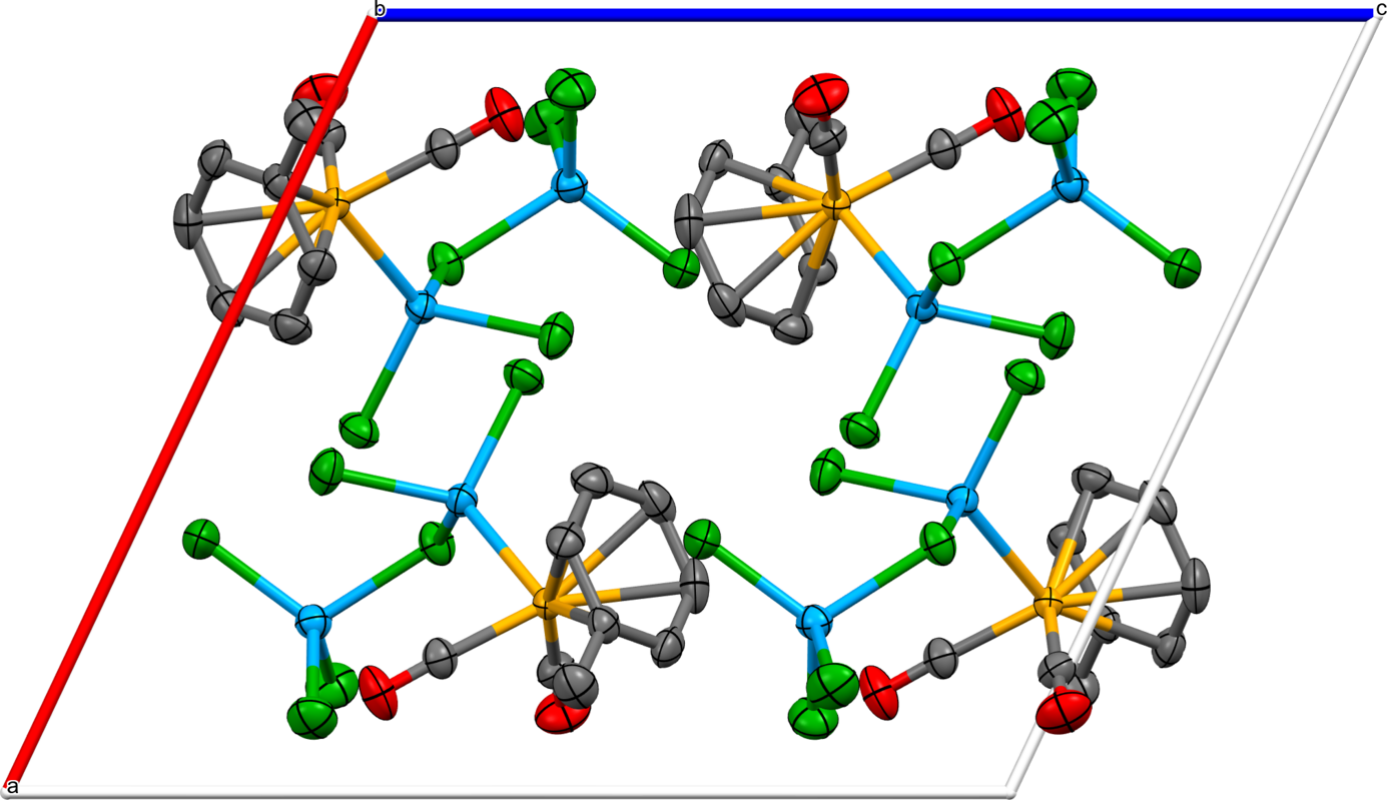
Crystal packing diagram viewed along the *b* axis. Hydrogen atoms have been omitted for clarity.

The Ru1—Ga1 bond length of 2.4575 (2) Å for the title compound is very similar to the value of 2.453 (1) Å observed for Ru_2_{GaCl_2_(THF)}_2_(CO)_8_ (Harakas & Whittlesey, 1997 ▸). The packing is shown in Fig. 2 ▸

During the work-up of the reaction, the title compound was isolated directly from the toluene solution. It is unknown at this time the role of di­phenyl­silanediol, if any, in the formation of the title compound. A solid that was insoluble in toluene in the reaction flask was extracted with THF forming an orange solution. This reaction product, which may contain the desired di­phenyl­siloxane metal cluster, has not yet been fully characterized.

## Synthesis and crystallization

All manipulations were carried out under argon using standard Schlenk line techniques. Our previous work (Demmin *et al.*, 2024 ▸) demonstrated that silicone-based vacuum grease can contaminate gallium halide reactions. Therefore, PTFE sleeves and non-silicone based vacuum grease were used on all glassware in this experiment. In a 250 ml Schlenk flask, gallium (5.60 g, 80.3 mmole) and GaCl_3_ 5.00 g (28.4, mmole) were combined followed by toluene (175 ml). The mixture was heated to reflux for 24 h to produce a solution containing gallium(II) chloride (Ga_2_Cl_4_), excess gallium was present.

Di­phenyl­silanediol (0.306 g, 1.41 mmol) was added to a 150 ml Schlenk flask followed by toluene (50 ml). To this flask, 10 ml of the Ga_2_Cl_4_ stock solution was added *via* cannula. The dark-gray mixture was refluxed under argon for 72 h resulting in a light-gray mixture. The reaction flask was cooled to 25°C and Ru_3_(CO)_12_ (0.225 g, 0.352 mmol) was then added. The mixture was heated to reflux for an additional 72 h. This resulted in a mixture with a suspended gray solid/gel and colorless solution. The colorless solution was deca­nted into a 150 ml Schlenk flask *via* cannula. After standing at 25°C for 10 days, colorless crystals were observed.

A single crystal was coated with NVH oil and mounted on a MiTeGen loop under a stream of argon gas then cooled to −25°C for data collection.

## Refinement

Crystal data, data collection, and structure refinement details are summarized in Table 2 ▸.

## Supplementary Material

Crystal structure: contains datablock(s) global, I. DOI: 10.1107/S2414314624006576/bt4152sup1.cif

Structure factors: contains datablock(s) I. DOI: 10.1107/S2414314624006576/bt4152Isup2.hkl

CCDC reference: 2367755

Additional supporting information:  crystallographic information; 3D view; checkCIF report

## Figures and Tables

**Table 1:** Selected geometric parameters (Å, °)

Ru1—Ga1	2.4575 (2)	Ga2—Cl6	2.1413 (6)
Ga1—Cl2	2.1665 (5)	Ga2—Cl4	2.1456 (6)
Ga1—Cl1	2.1888 (5)	Ga2—Cl5	2.1521 (6)
Ga1—Cl3	2.4619 (5)	Ga2—Cl3	2.2583 (5)
			
Cl6—Ga2—Cl4	114.95 (3)	Cl6—Ga2—Cl3	110.96 (2)
Cl6—Ga2—Cl5	110.36 (3)	Cl4—Ga2—Cl3	106.43 (2)
Cl4—Ga2—Cl5	109.67 (3)	Cl5—Ga2—Cl3	103.82 (2)

**Table 2:** Experimental details

Crystal data	
Chemical formula	[RuGa_2_Cl_6_(C_7_H_8_)(CO)_2_]
*M*_r_	601.36
Crystal system, space group	Monoclinic, *P*2_1_/*c*
Temperature (K)	248
*a*, *b*, *c* (Å)	13.1598 (6), 9.7142 (4), 15.3369 (7)
β (°)	115.257 (1)
*V* (Å^3^)	1773.19 (14)
*Z*	4
Radiation type	Mo *K*α
μ (mm^−1^)	4.76
Crystal size (mm)	0.36 × 0.34 × 0.31

Data collection	
Diffractometer	Bruker D8 Quest Eco, Photon II 7
Absorption correction	Multi-scan (*SADABS*; Krause *et al.*, 2015 ▸)
*T*_min_, *T*_max_	0.21, 0.32
No. of measured, independent and observed [*I* > 2σ(*I*)] reflections	66270, 4424, 4133
*R*_int_	0.031
(sin θ/λ)_max_ (Å^−1^)	0.668

Refinement	
*R*[*F*^2^ > 2σ(*F*^2^)], *wR*(*F*^2^), *S*	0.016, 0.041, 1.09
No. of reflections	4424
No. of parameters	182
H-atom treatment	H-atom parameters constrained
Δρ_max_, Δρ_min_ (e Å^−3^)	0.65, −0.43

Computer programs: *APEX3* and *SAINT* (Bruker, 2019 ▸), *SHELXT2018/2* (Sheldrick, 2015*a* ▸), *SHELXL2018/3* (Sheldrick, 2015*b* ▸) and *ShelXle* (Hübschle *et al.*, 2011 ▸).

## full crystallographic data

### Dicarbonyl-1κ2C-µ-chlorido-2:3κ2Cl:Cl-pentachlorido-2κ2Cl,3κ3Cl-[1(η6)-toluene]digallium(III)ruthenium(I)(Ru—Ga) Crystal data

**Table:**

[RuGa_2_Cl_6_(C_7_H_8_)(CO)_2_]	*F*(000) = 1144
*M**_r_* = 601.36	*D*_x_ = 2.253 Mg m^−^^3^
Monoclinic, *P*2_1_/*c*	Mo *K*α radiation, λ = 0.71073 Å
*a* = 13.1598 (6) Å	Cell parameters from 9595 reflections
*b* = 9.7142 (4) Å	θ = 2.7–28.3°
*c* = 15.3369 (7) Å	µ = 4.76 mm^−^^1^
β = 115.257 (1)°	*T* = 248 K
*V* = 1773.19 (14) Å^3^	Block, clear colourless
*Z* = 4	0.36 × 0.34 × 0.31 mm

### Dicarbonyl-1κ2C-µ-chlorido-2:3κ2Cl:Cl-pentachlorido-2κ2Cl,3κ3Cl-[1(η6)-toluene]digallium(III)ruthenium(I)(Ru—Ga) Data collection

**Table:**

Bruker D8 Quest Eco, Photon II 7 diffractometer	4133 reflections with *I* > 2σ(*I*)
Detector resolution: 7.3910 pixels mm^-1^	*R*_int_ = 0.031
phi and ω scans	θ_max_ = 28.3°, θ_min_ = 2.7°
Absorption correction: multi-scan (SADABS; Krause *et al.*, 2015)	*h* = −17→17
*T*_min_ = 0.21, *T*_max_ = 0.32	*k* = −12→12
66270 measured reflections	*l* = −20→20
4424 independent reflections	

### Dicarbonyl-1κ2C-µ-chlorido-2:3κ2Cl:Cl-pentachlorido-2κ2Cl,3κ3Cl-[1(η6)-toluene]digallium(III)ruthenium(I)(Ru—Ga) Refinement

**Table:**

Refinement on *F*^2^	0 restraints
Least-squares matrix: full	Hydrogen site location: inferred from neighbouring sites
*R*[*F*^2^ > 2σ(*F*^2^)] = 0.016	H-atom parameters constrained
*wR*(*F*^2^) = 0.041	*w* = 1/[σ^2^(*F*_o_^2^) + (0.0179*P*)^2^ + 1.0566*P*] where *P* = (*F*_o_^2^ + 2*F*_c_^2^)/3
*S* = 1.09	(Δ/σ)_max_ = 0.002
4424 reflections	Δρ_max_ = 0.65 e Å^−^^3^
182 parameters	Δρ_min_ = −0.43 e Å^−^^3^

### Dicarbonyl-1κ2C-µ-chlorido-2:3κ2Cl:Cl-pentachlorido-2κ2Cl,3κ3Cl-[1(η6)-toluene]digallium(III)ruthenium(I)(Ru—Ga) Special details

**Table:**

Geometry. All esds (except the esd in the dihedral angle between two l.s. planes) are estimated using the full covariance matrix. The cell esds are taken into account individually in the estimation of esds in distances, angles and torsion angles; correlations between esds in cell parameters are only used when they are defined by crystal symmetry. An approximate (isotropic) treatment of cell esds is used for estimating esds involving l.s. planes.

### Dicarbonyl-1κ2C-µ-chlorido-2:3κ2Cl:Cl-pentachlorido-2κ2Cl,3κ3Cl-[1(η6)-toluene]digallium(III)ruthenium(I)(Ru—Ga) Fractional atomic coordinates and isotropic or equivalent isotropic displacement parameters (Å^2^)

**Table:**

	*x*	*y*	*z*	*U*_iso_*/*U*_eq_	
Ru1	0.24301 (2)	0.66776 (2)	0.55005 (2)	0.02484 (4)	
Ga1	0.37587 (2)	0.52947 (2)	0.68455 (2)	0.02795 (5)	
Ga2	0.21861 (2)	0.26035 (2)	0.77462 (2)	0.03228 (5)	
Cl1	0.53434 (4)	0.47105 (6)	0.68030 (4)	0.04053 (11)	
Cl2	0.41255 (5)	0.58077 (6)	0.83247 (3)	0.04424 (12)	
Cl3	0.31993 (4)	0.28855 (5)	0.68851 (4)	0.03697 (10)	
Cl4	0.09559 (5)	0.42180 (6)	0.73078 (5)	0.05227 (13)	
Cl5	0.13673 (5)	0.06499 (6)	0.72620 (5)	0.05478 (14)	
Cl6	0.32572 (4)	0.25357 (7)	0.92593 (4)	0.05277 (15)	
O1	0.12790 (15)	0.73771 (17)	0.67684 (13)	0.0519 (4)	
O2	0.10336 (14)	0.40984 (18)	0.48352 (13)	0.0573 (4)	
C1	0.17226 (16)	0.7071 (2)	0.63130 (14)	0.0338 (4)	
C2	0.15713 (16)	0.5047 (2)	0.51002 (14)	0.0367 (4)	
C3	0.1370 (2)	1.0026 (3)	0.48306 (18)	0.0502 (5)	
H3A	0.151190	1.085031	0.454310	0.075000*	
H3B	0.148544	1.022284	0.548647	0.075000*	
H3C	0.060080	0.972800	0.445748	0.075000*	
C4	0.21607 (15)	0.8907 (2)	0.48377 (13)	0.0324 (4)	
C5	0.18667 (17)	0.7958 (2)	0.40924 (13)	0.0389 (4)	
H5	0.107892	0.790389	0.359964	0.047000*	
C6	0.2656 (2)	0.6935 (3)	0.41157 (15)	0.0478 (5)	
H6	0.241232	0.618942	0.362932	0.057000*	
C7	0.37233 (19)	0.6881 (2)	0.48817 (17)	0.0458 (5)	
H7	0.423488	0.610611	0.493398	0.055000*	
C8	0.40090 (17)	0.7834 (2)	0.56321 (16)	0.0432 (5)	
H8	0.471964	0.772111	0.622005	0.052000*	
C9	0.32405 (16)	0.8811 (2)	0.56150 (14)	0.0371 (4)	
H9	0.340902	0.937264	0.619916	0.044000*	

### Dicarbonyl-1κ2C-µ-chlorido-2:3κ2Cl:Cl-pentachlorido-2κ2Cl,3κ3Cl-[1(η6)-toluene]digallium(III)ruthenium(I)(Ru—Ga) Atomic displacement parameters (Å^2^)

**Table:**

	*U*^11^	*U*^22^	*U*^33^	*U*^12^	*U*^13^	*U*^23^
Ru1	0.02522 (6)	0.02903 (7)	0.02071 (6)	−0.00104 (5)	0.01024 (5)	0.00055 (5)
Ga1	0.02861 (9)	0.03056 (10)	0.02508 (9)	0.00314 (7)	0.01183 (7)	0.00206 (7)
Ga2	0.03015 (10)	0.03326 (11)	0.03336 (10)	−0.00027 (8)	0.01347 (8)	0.00417 (8)
Cl1	0.0333 (2)	0.0472 (3)	0.0458 (3)	0.00639 (19)	0.0213 (2)	0.0039 (2)
Cl2	0.0559 (3)	0.0468 (3)	0.0281 (2)	0.0091 (2)	0.0161 (2)	−0.00377 (19)
Cl3	0.0476 (2)	0.0293 (2)	0.0424 (2)	−0.00163 (19)	0.0273 (2)	0.00116 (18)
Cl4	0.0467 (3)	0.0490 (3)	0.0610 (3)	0.0157 (2)	0.0228 (3)	0.0080 (3)
Cl5	0.0554 (3)	0.0405 (3)	0.0623 (3)	−0.0155 (2)	0.0192 (3)	0.0040 (3)
Cl6	0.0389 (2)	0.0864 (4)	0.0323 (2)	0.0041 (3)	0.0145 (2)	0.0060 (3)
O1	0.0721 (11)	0.0459 (9)	0.0607 (10)	0.0051 (8)	0.0503 (9)	0.0003 (8)
O2	0.0500 (9)	0.0449 (9)	0.0626 (10)	−0.0152 (8)	0.0103 (8)	−0.0092 (8)
C1	0.0408 (9)	0.0295 (9)	0.0349 (9)	0.0002 (7)	0.0198 (8)	0.0036 (7)
C2	0.0334 (9)	0.0387 (10)	0.0334 (9)	−0.0019 (8)	0.0099 (7)	−0.0010 (8)
C3	0.0545 (13)	0.0464 (12)	0.0567 (14)	0.0116 (10)	0.0304 (11)	0.0103 (10)
C4	0.0365 (9)	0.0339 (9)	0.0302 (8)	−0.0027 (7)	0.0175 (7)	0.0051 (7)
C5	0.0422 (10)	0.0474 (11)	0.0233 (8)	−0.0055 (9)	0.0101 (7)	0.0060 (8)
C6	0.0754 (15)	0.0475 (12)	0.0321 (10)	−0.0045 (11)	0.0342 (11)	−0.0027 (9)
C7	0.0519 (12)	0.0497 (12)	0.0532 (12)	0.0122 (10)	0.0392 (11)	0.0147 (10)
C8	0.0308 (9)	0.0520 (12)	0.0473 (11)	−0.0053 (9)	0.0172 (8)	0.0101 (10)
C9	0.0371 (9)	0.0378 (10)	0.0343 (9)	−0.0111 (8)	0.0133 (8)	0.0000 (8)

### Dicarbonyl-1κ2C-µ-chlorido-2:3κ2Cl:Cl-pentachlorido-2κ2Cl,3κ3Cl-[1(η6)-toluene]digallium(III)ruthenium(I)(Ru—Ga) Geometric parameters (Å, º)

**Table:**

Ru1—C1	1.8856 (19)	Ga2—Cl4	2.1456 (6)
Ru1—C2	1.890 (2)	Ga2—Cl5	2.1521 (6)
Ru1—C6	2.2803 (19)	Ga2—Cl3	2.2583 (5)
Ru1—C7	2.2834 (19)	O1—C1	1.125 (2)
Ru1—C8	2.294 (2)	O2—C2	1.127 (3)
Ru1—C9	2.3027 (19)	C3—C4	1.502 (3)
Ru1—C5	2.3223 (19)	C4—C5	1.389 (3)
Ru1—C4	2.3536 (19)	C4—C9	1.415 (3)
Ru1—Ga1	2.4575 (2)	C5—C6	1.426 (3)
Ga1—Cl2	2.1665 (5)	C6—C7	1.396 (3)
Ga1—Cl1	2.1888 (5)	C7—C8	1.398 (3)
Ga1—Cl3	2.4619 (5)	C8—C9	1.379 (3)
Ga2—Cl6	2.1413 (6)		
			
C1—Ru1—C2	89.37 (9)	Cl2—Ga1—Cl1	107.54 (2)
C1—Ru1—C6	152.94 (9)	Cl2—Ga1—Ru1	120.934 (17)
C2—Ru1—C6	94.99 (9)	Cl1—Ga1—Ru1	117.546 (16)
C1—Ru1—C7	157.35 (9)	Cl2—Ga1—Cl3	97.36 (2)
C2—Ru1—C7	112.70 (9)	Cl1—Ga1—Cl3	93.035 (19)
C6—Ru1—C7	35.62 (9)	Ru1—Ga1—Cl3	115.292 (15)
C1—Ru1—C8	121.78 (9)	Cl6—Ga2—Cl4	114.95 (3)
C2—Ru1—C8	146.39 (9)	Cl6—Ga2—Cl5	110.36 (3)
C6—Ru1—C8	63.59 (9)	Cl4—Ga2—Cl5	109.67 (3)
C7—Ru1—C8	35.56 (9)	Cl6—Ga2—Cl3	110.96 (2)
C1—Ru1—C9	96.35 (8)	Cl4—Ga2—Cl3	106.43 (2)
C2—Ru1—C9	166.51 (8)	Cl5—Ga2—Cl3	103.82 (2)
C6—Ru1—C9	74.46 (8)	Ga2—Cl3—Ga1	112.77 (2)
C7—Ru1—C9	63.31 (8)	O1—C1—Ru1	175.86 (18)
C8—Ru1—C9	34.91 (8)	O2—C2—Ru1	177.7 (2)
C1—Ru1—C5	117.03 (8)	C5—C4—C9	118.47 (18)
C2—Ru1—C5	103.73 (8)	C5—C4—C3	121.72 (19)
C6—Ru1—C5	36.09 (8)	C9—C4—C3	119.81 (19)
C7—Ru1—C5	64.28 (8)	C5—C4—Ru1	71.49 (11)
C8—Ru1—C5	74.78 (7)	C9—C4—Ru1	70.35 (11)
C9—Ru1—C5	62.79 (7)	C3—C4—Ru1	130.19 (13)
C1—Ru1—C4	94.20 (7)	C4—C5—C6	119.83 (19)
C2—Ru1—C4	132.21 (8)	C4—C5—Ru1	73.96 (11)
C6—Ru1—C4	63.40 (8)	C6—C5—Ru1	70.35 (11)
C7—Ru1—C4	75.08 (7)	C7—C6—C5	120.5 (2)
C8—Ru1—C4	63.34 (7)	C7—C6—Ru1	72.31 (11)
C9—Ru1—C4	35.37 (7)	C5—C6—Ru1	73.56 (11)
C5—Ru1—C4	34.55 (7)	C6—C7—C8	119.2 (2)
C1—Ru1—Ga1	86.05 (6)	C6—C7—Ru1	72.07 (12)
C2—Ru1—Ga1	85.82 (6)	C8—C7—Ru1	72.63 (11)
C6—Ru1—Ga1	120.86 (7)	C9—C8—C7	120.2 (2)
C7—Ru1—Ga1	90.38 (6)	C9—C8—Ru1	72.89 (11)
C8—Ru1—Ga1	84.29 (6)	C7—C8—Ru1	71.81 (12)
C9—Ru1—Ga1	106.70 (5)	C8—C9—C4	121.73 (19)
C5—Ru1—Ga1	154.66 (5)	C8—C9—Ru1	72.20 (12)
C4—Ru1—Ga1	141.95 (5)	C4—C9—Ru1	74.28 (11)
